# Feasibility of exercise therapy for children with asthma: a meta-analysis

**DOI:** 10.3389/fcell.2023.1192929

**Published:** 2023-07-10

**Authors:** Linyuan Zhou, Haofu Xu

**Affiliations:** Special Operation College of PLA, Guangzhou, China

**Keywords:** asthma, exercise therapy, children, lung function, immune system

## Abstract

**Background:** Although numerous studies have demonstrated the clear benefits of exercise for people with asthma, controversy remains. This study evaluated the effects of sustained exercise types on lung function and quality of life in patients with asthma.

**Methods:** We searched PubMed, EMBASE, Web of Science, Cochrane Library, China National Knowledge Infrastructure (CNKI), and Wanfang database since January 2000 to August 2022 .included randomized controlled trials (RCTs) of asthmatic children intervened with exercise. The outcomes were lung function and asthma-related quality of life. Fixed-effects model (I2≤50%) or random-effects model (I2>50%) was applied to calculate the pooled effects. Funnel plots were quantified to present publication bias, and a P value <0.05 was statistically significant.

**Results:** Eventually, 15 trials conformed to the selection criteria. The exercise group significantly improved lung function (FEV1 and FVC) in asthmatic children compared with the control group. Forced Expiratory Volume in 1 Second (MD = 2.12, 95%CI = 0.70, 3.53; *p* = 0.003; I^2^ = 15%); Forced Vital Capacity (MD = 2.78, 95%CI = 1.26, 4.31; *p* = 0.0004; I^2^ = 56%). The immune system markers IL-6 and TNF-α, were significantly reduced in the exercise group. Interleukin-6 (MD = −0.49, 95%CI = −0.81, −0.17; *p* = 0.003; I^2^=0%); tumor necrosis factor-α (MD = −0.54, 95%CI = −0.92, −0.15; *p* = 0.006; I^2^ = 0%). That quality of life (PAQLQ) was significantly improved in children with asthma in the exercise group. PAQLQ-Total score (MD = 1.06, 95%CI = 0.46, 1.66; *p* = 0.006; I^2^ = 94%); PAQLQ-Emotional (MD = 0.91, 95%CI = 0.76, 1.06; *p*<0.00001; I^2^ = 90%); PAQLQ-symptoms (MD = 0.87, 95%CI = 0.71, 1.02; *p*<0.00001; I^2^ = 95%); PAQLQ-activities (MD = 1.20, 95%CI = 0.58, 1.82; *p* = 0.00001; I^2^ = 93%). Meta-analysis showed significant improvements in body composition in the exercise group. BMI (MD = −2.42, 95%CI = −4.40, 0.44; *p* = 0.02; I^2^ = 85%).

**Conclusions:** This meta-analysis demonstrated the effectiveness of exercise in improving pulmonary function index (FEV1, FVC), immune system (IL-6, TNF-α, Feno), exercise ability (6MWT), body composition (BMI), and quality of life (PAQLQ) in asthmatic children. Asthmatic children should regularly participate in physical exercise.

## 1 Introduction

Asthma is one of the most common chronic diseases among adults and children all around the world. Asthma is a heterogeneous disease characterized by chronic airway inflammation and airway hyperresponsiveness. The chronic inflammation causes associated airway hyperresponsiveness that leads to recurrent wheezing, cough, breathlessness, and chest tightness, which often triggers attacks or exacerbates at night and in the early morning. Around 300 million people of all ages experience asthma and related complications, and about 250,000 people die from asthma each year (Bousquet and Khaltaev, 2007). Experts predict that 100 million people will still live with asthma in 2025. The prevalence of childhood asthma in China soared from 1.09% in 1990 to 3.02% in 2010 (BAO Yixiao et al., 2016). However, standardized treatment of asthma is mature and has been used globally for many years. With the introduction of the GINA program and the implementation of the guidelines for the prevention and treatment of childhood asthma in China, the early diagnosis and management of childhood asthma in Chinese pediatricians have been enhanced. However, the overall control of childhood asthma is still poor (Sha Li et al., 2016). The Third National Childhood Asthma Epidemiological Survey in China showed that 77% of children with asthma had an acute episode in the past year (LIU Chuanhe et al., 2013). Medication has long been the primary means of asthma control, but physical therapy is another effective means. Physical therapy mainly includes exercise therapy, respiratory exercise, and muscle training (Wang et al., 2019).

Although some scholars have conducted systematic reviews and meta-analyses on the effects of physical therapy on childhood asthma, these scholars did not exclude adults in the research process. Also, they lumped together three different physical therapy methods: exercise therapy, respiratory exercise and respiratory muscle training (Wang et al., 2019; hang et al., 2021; YANG Yiyun et al., 2021). Compared with respiratory exercise and respiratory muscle training, exercise therapy is more social and recreational, and exercise therapy can better promote children’s physical development. Exercise therapy has also been shown as safe and effective, and the benefits of regular exercise are significant for children with asthma (Lang, 2019). Therefore, this meta-analysis only focused on children and excluded respiratory exercise and muscle training. This study aimed to show the effects of different exercise intensity, no exercise modes, and other exercise intervention times on children’s lung function and quality of life and to provide recommendations and references for pediatricians to treat and control children’s asthma using exercise therapy.

## 2 Methods

This meta-analysis was performed following the statement of the Preferred Reporting Items for Systematic Review and Meta-Analysis (PRISMA) guidelines (Moher et al., 2009) in order to provide comprehensive and transparent reporting of methods and results.

### 2.1 Search strategy

**FIGURE 1:**
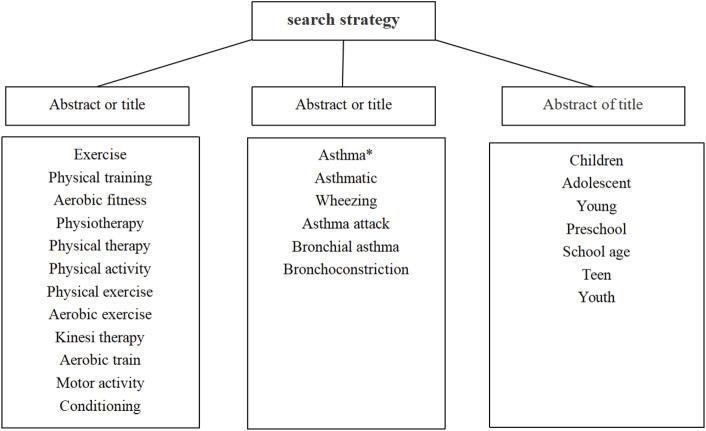
A search strategy for English database using the following keywords b and search strategy using the following keywords to search Chinese databases.

Electronic article searches were conducted between January 2000 and August 2022 in PubMed, Cochrane Library, Web of Science, EMBASE, China National Knowledge Infrastructure (CNKI), and Wanfang database. The language of articles was English and Chinese. The search terms in this review were a combination of exercise intervention terms AND asthma related terms AND adolescent population terms. Figure 1 provides the keywords and search strategy. Three researchers independently screened titles, abstracts, and full-text articles of potentially relevant studies. We further searched for additional relevant articles in Google Scholar and identified potential references for inclusion from previous systematic review and meta-analyses.

### 2.2 Eligibility criteria

The inclusion criteria for studies are as follows: 1) Participants: Participants should have asthma diagnosed by well-defined or internationally recognized criteria, and they should not be older than 18 or younger than 6, regardless of gender, ethnicity, or region. 2) Intervention type: We included studies with an exercise training intervention in children with asthma, and the intervention period less than 2 weeks were acceptable. 3) Outcome: At least one of the four main outcomes (lung function, airway inflammation, exercise ability, and health and life quality) had to be reported to be included. The specific outcome measures of lung function were Forced Vital Capacity (FVC), Forced Expiratory Volume in 1 Second (FEV1), and Peak Expiratory Flow (PEF). The exercise capacity outcome measure was six walk minute test (6WMT). The airway inflammation indicators were tumor necrosis factor-α (TNF-α), Interleukin- 6 (IL-6), and Fractional exhaled nitric oxide (FeNO). The health and life quality outcomes were Pediatric Asthma Quality of Life Questionnaire (PAQLQ) and Body Mass Index (BMI). 4) Study design: We included randomized controlled trials that compared exercise training interventions with no intervention.

### 2.3 Studies screening and data extraction

Two authors independently evaluated all studies for potential eligibility. In the case of disagreement, a third party adjudicated whether a study met the inclusion criteria. The documents were transferred to a third researcher for adjudication if they disagreed. For each study, data were extracted regarding the first author’s last name, year of publication, country, participants characteristics (mean age, sample size), exercise intervention (type, duration, frequency, intensity), and the mean values of two time points (pre- and post-intervention) with corresponding standard deviations. The outcome measures were: FVC, FeV1, PEF, 6WMT, TNF-α, IL-6, FeNO, BMI, PAQLQ-Total score, PAQLQ-Emotional score, PAQLQ-symptoms score, and PAQLQ-activities score. When there was insufficient information the research team attempted to contact the corresponding author. Tables 1, 2 provides detailed information on these studies.

**TABLE 1:** Exercise Program details of eligible studies.

Author	Years	Exercise	Duration (weeks) x Frequency(d/wk)	Time (minutes)	Intensity
Winn	2021	HIIT	16x3	30	90%HR_max_
Qiong Chen	2020	Aerobic Exercise	12x3	40-60	1∼4wk50–60%HR_max_ 5∼12wk60–70%HR_max_
Jing Tan	2019	Exercise	12x3	20-30	No
Abdelbasset	2018	Aerobic Exercise	10x3	40	50–70%HR_max_
Carew	2017	Swimming,Football And Basketball	6x1	40	No
Jia Li	2016	HIIT and Aerobic exercise	8x3	40	90%VO_2peak_ and 50%VO_2peak_
Latorre-Roman	2014	Combined Training	12x3	60	No
Andrade	2014	Aerobic Exercise	6x3	1∼2wk 20 3∼6wk 30	70–80%HR_max_
Kader	2013	Aerobic Exercise	8X4	25-45	60–80%HR_max_
Onur	2011	Aerobic Exercise	8x2	45	80%HR_max_
Wicher	2010	Swimming	12x2	60	No
JengShing Wang	2009	Swimming	6x3	50	No
Basaran	2006	Basketball	8x3	60	No
Counil	2003	Aerobic Exercise	6x3	45	No
Weisgerber	2003	Swimming	5-6x3	45	No

**TABLE 2:** Demographics and outcomes.

Author	years	Participants	Age (years)	Gender	BMI	Overcomes				
						Lung function	Athletic ability	Immune system	Body composition	Quality of life
Winn	2021	E(29) C(68)	E(13.1±1.0) C(12.9±1.2)	78M & 77F	22.7±4.7	FEV1% FVC% PEF%	No	FeNO	BMI	PAQLQ
Qiong Chen	2020	E(20) C(20)	E(11.4 ± 2.8) C(12.1 ± 3.4)	14M & 26F	No	FVC% FEV1% PEF%	6MWT	No	No	PAQLQ
Jing Tan	2019	E(80) C(76)	E(8.2 ± 2.1) C(8.1 ± 2.1)	97M & 59F	No	FVC% FEV1% PEF%	No	No	No	No
Abdelbasset	2018	E(19) C(19)	E(9.8 ± 2.3) C(11.7 ± 2.3)	23M & 15F	21.3±3.0	FEV1% FVC%	6MWT	No	BMI	PAQLQ
Carew	2017	E(29) C(12)	E(13.5 ± 1.8) C(12.0 ± 3.1)	24M & 17F	No	FVC% FEV1% PEF%	No	No	No	No
Jia Li	2016	E(29) C(12)	E(12.5 ± 1.6) C(10.8 ± 1.3)	26M & 15F	18.3±2.0 18.2±3.2	FVC% FEV1% PEF%	6MWT	IL-6 TNF-α FeNO	BMI	No
Latorre-Roman	2014	E(58) C(47)	E(11.5 ± 1.0) C(11.5 ± 1.4)	Not Described	17.49±2.8	No	6MWT	No	BMI	PAQLQ
Andrade	2014	E(10) C(17)	E(11.7 ± 2.3) C(11.4 ± 2.3)	15M & 12F	No	No	6MWT	No	No	PAQLQ
Kader	2013	E(40) C(40)	E(13.2 ± 3.5) C(12.6 ± 3.2)	42M & 38F	27.15 ± 2.38	No	No	IL-6 TNF-α	BMI	No
Onur	2011	E(30) C(13)	E(9.8 ± 1.8) C(10.3 ± 2.0)	28M & 15F	No	FEV1% FVC%	No	No	No	No
Wicher	2010	E(30) C(31)	E(10.4 ± 3.1) C(10.9 ± 2.6)	27M & 34F	No	FEV1% FVC%	No	No	No	No
JengShing Wang	2009	E(15) C(15)	E(9-11) C(9-11)	20M & 10F	No	FEV1% FVC%	No	No	No	No
Basaran	2006	E(31) C(31)	E(10.4 ± 2.2) C(10.5 ± 2.1)	40M & 22F	No	FEV1% FVC% PEF%	6MWT	No	No	PAQLQ
Counil	2003	E(7) C(7)	E(13.9 ± 0.8) C(14.0 ± 0.6)	14M	No	FEV1%	No	No	No	No
Weisgerber	2003	E(5) C(3)	E(8.4 ± 1.5) C(7.3 ± 0.6)	4M & 4F	No	FEV1% FVC% PEF%	No	No	No	No

FEV1, Forced Expiratory Volume in 1 Second; FVC, Forced Vital Capacity; PEF, Peak Expiratory Flow; 6MWT, 6-min walking test; IL-6, Interleukin- 6; TNF-α tumor necrosis factor-α; FeNO, Fractional Exhaled Nitric Oxide; BMI, Body Mass Index; PAQLQ, Paediatric Asthma Quality of Life Questionnaire.

### 2.4 Study selection

**FIGURE 2:**
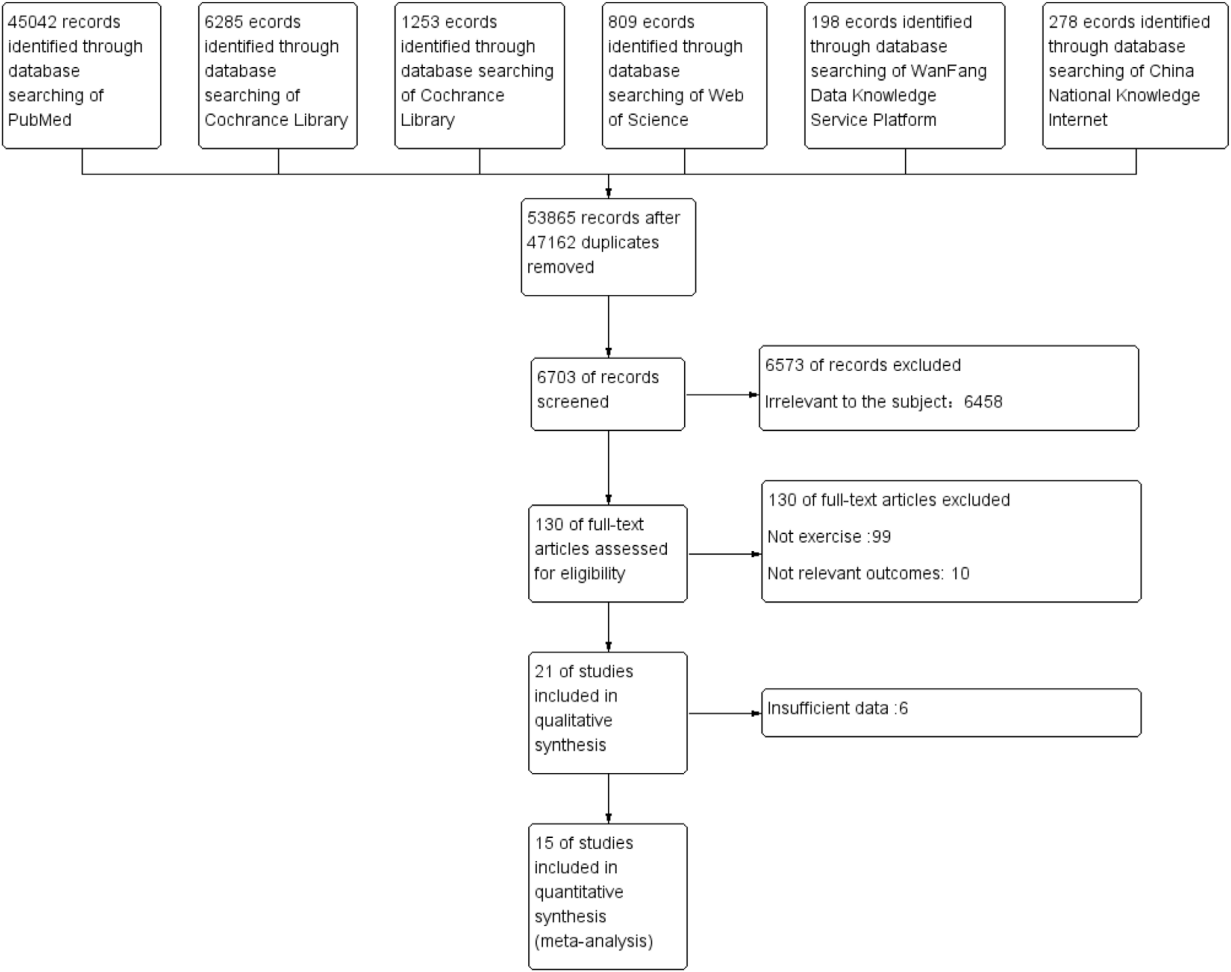
Search screening process.

We searched identified 53,865 articles, including 45,042 articles from PubMed, 1,253 articles from the Cochrane Library studies, 6,285 articles from the Embase studies, 809 articles from the Web of Science studies, 198 articles from the WanFang, and 278 articles from the CNKI.47,162 duplicate articles eliminated. By reading the title and abstract, six thousand five hundred seventy-three irrelevant documents were removed. There were 130 to read, 115 to delete, and 15 included in the Meta-analysis. Figure 2 shows a flow chart of the search screening process (Counil et al., 2003; Weisgerber et al., 2003; Basaran et al., 2006; WANG and HUNG, 2009; Wicher et al., 2010; Onur et al., 2011; El-Kader et al., 2013; de Andrade et al., 2014; Latorre-Román et al., 2014; Jia et al., 2016; Abdelbasset et al., 2018; Carew and Cox, 2018; Jing et al., 2019; Chen et al., 2020; Winn et al., 2021).

### 2.5 Risk of bias in studies

Two reviewers independently assessed each included study using the Cochrane risk of bias tool. For RCTs, the Cochrane Collaboration Guideline specifies six domains, including bias arising from the randomization process, bias owing to deviations from intended interventions, bias owing to missing outcome data, bias in measurement of the outcome, bias in selection of the reported, and other biases (Higgins et al., 2011). Risk of bias was stated as “low risk,” “high risk,” or “unclear.”

Risk of bias are provided in Table 2. 6/15 studies described the randomization method of the subjects in detail. 2/15 studies described the allocation concealment scheme of the subjects in detail. 2/15 studies blinded patients and experimenters, and 3/15 studies blinded outcome evaluators. 10/15 studies reported the integrity of the results in detail, 1 study did not report the number of lost visits in detail, and 4 studies had too many lost visits, which affected data integrity. 13/15 studies reported the results of the data in detail, and 2 studies did not report the results. There was no other bias in 14/15 studies.

### 2.6 Publication bias

**FIGURE 3:**
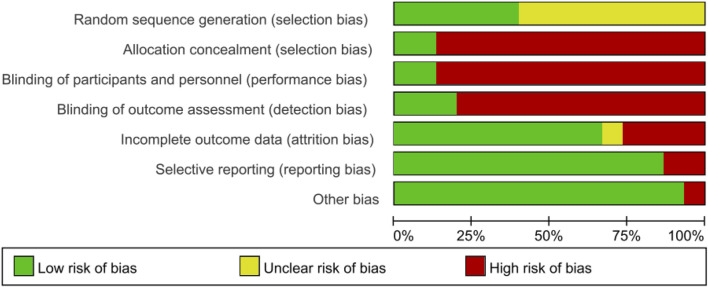
Overall risk bias evaluation.

**FIGURE 4:**
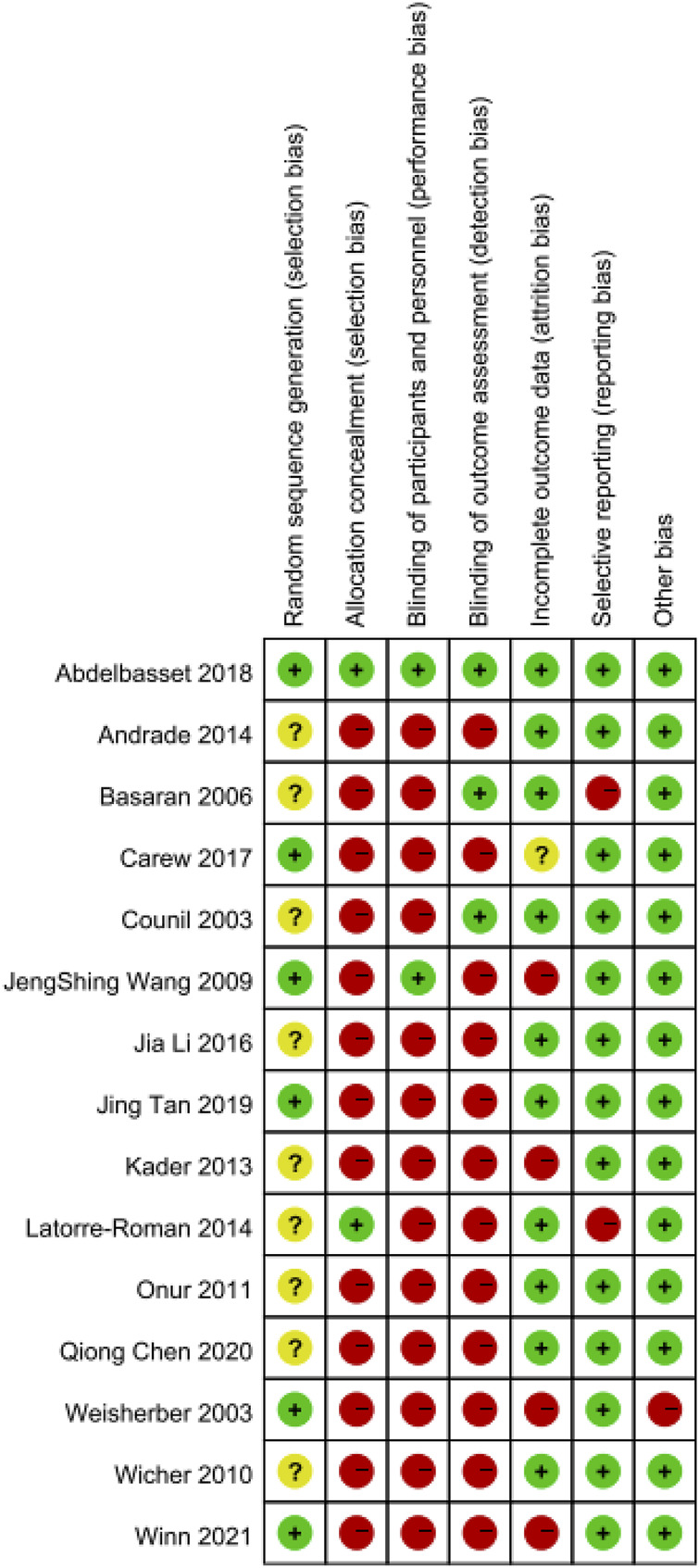
Overall risk bias summary.

Publication bias was assessed by funnel plot and Egger test. First, funnel plot was drawn by Stata 17 software, and then Egger test was carried out. When *p*>0.05, there was no publication bias. The risk of bias is summarized in Figure 3 and Figure 4.

### 2.7 Statistical analysis

A meta-analysis was performed using ReMan software (version 5.3) to evaluate the effect of exercise intervention on children with asthma. Effect sizes for main outcomes are expressed as standardized mean differences (SMD). If the standard deviation was not reported, it was estimated from the reported standard error, the 95% confidence interval, interquartile range or *p*-value related to the pertinent number of participants. To better interpret SMD, we used Hedges “gas small (0.2), medium (0.5) and large (0.8) (Cohen, 2013).” The heterogeneity between trial results was tested for I2 statistics. I2 values of <25%, 25%–50%, and >50% are acknowledged to represent small, medium, and large amounts of heterogeneity (Higgins and Thompson, 2002).

If heterogeneity is greater than 50%, a sensitivity analysis will be performed. Moreover, two analyses with subgroups were studied: exercise type (HIIT, Swimming, Ball, Aerobic exercise) and frequency of the exercise intervention (≤8 weeks vs. > 8 weeks).

## 3 Meta-analysis

### 3.1 Effects of exercise on lung function in children with asthma

**FIGURE 5:**
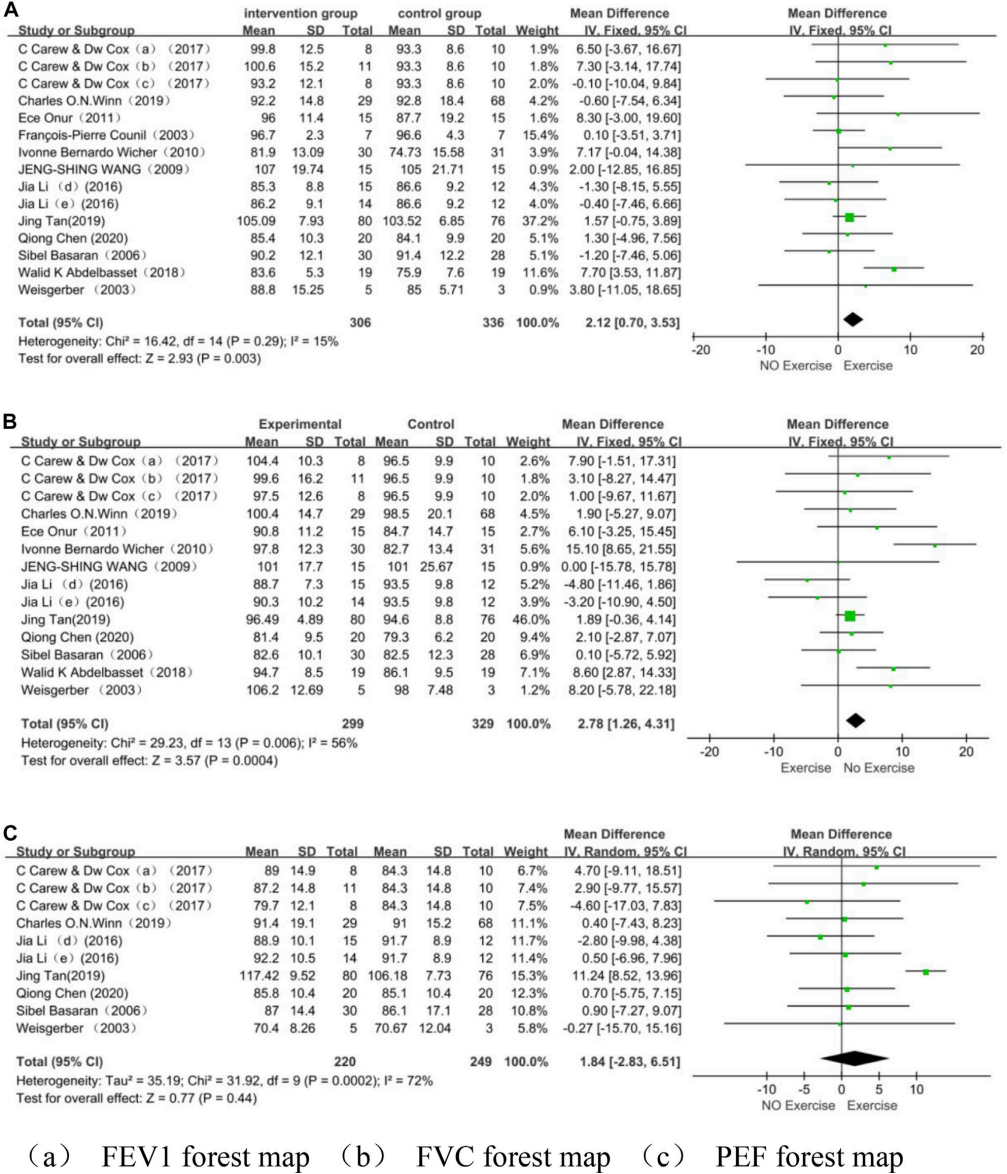
**(A)** FEV1 forest map **(B)** FVC forest map **(C)** PEF forest map.

Meta-analysis showed that the exercise group significantly improved lung function (FEV1 and FVC) in asthmatic children compared with the control group, but exercise did not improve PEF alone significantly. Forced Expiratory Volume in 1 Second (MD = 2.12, 95% CI = 0.70, 3.53; *p* = 0.003; I^2^ = 15%); Forced Vital Capacity (MD = 2.78, 95% CI = 1.26, 4.31; *p* = 0.0004; I^2^ = 56%), Peak Expiratory Flow (MD = 1.84, 95% CI = −2.83, 6.51; *p* = 0.44; I^2^ = 72%) (Figure 5).

The results of the subgroup analysis showed that aerobic exercise and swimming significantly improved lung function (FEV1 and FVC) in children with asthma and that HIIT and ball exercise improvements were not significant. Exercise intervention of over 8 weeks significantly improved lung function (FEV1 and FVC) in children with asthma, and exercise intervention effects of less than 8 weeks were not significant. The exercise was insignificant in children with asthma (PEF) (Table 4).

Effects of different exercise modes on FEV1 in asthmatic children. Swimming (MD = 5.98, 95% CI = 0.85, 11.11; *p* = 0.02; I^2^ = 0%); Aerobic exercise (MD = 2.89, 95% CI = 0.58, 5.19; *p* = 0.01; I2 = 57%); Ball (MD = 0.79, 95% CI = −3.94, 5.51; *p* = 0.74; I^2^ = 0%); HIIT (MD = −0.95, 95% CI = 5.83, 3.92; *p* = 0.70; I^2^ = 0%). Effects of different exercise modes on FVC in asthmatic children. Swimming (MD = 11.07, 95% CI = 6.33, 15.82; *p* < 0.00001; I^2^ = 24%); Aerobic exercise (MD = 5.06, 95% CI = 1.57, 8.54; *p* = 0.004; I2 = 30%); Ball (MD = 0.77, 95% CI = −3.88, 5.43; *p* = 0.74; I^2^ = 0%); HIIT (MD = 0.80, 95% CI = −4.28, 5.89; *p* = 0.76; I^2^ = 46%). Effects of different exercise modes on PEF in asthmatic children: Swimming (MD = 2.49, 95% CI = −7.80, 12.78; *p* = 0.64; I^2^ = 0%); Aerobic exercise (MD = 0.61, 95% CI = 4.26, 5.49; *p* = 0.8; I^2^ = 0%); Ball (MD = 0.06, 95% CI = −5.94, 6.07; *p* = 0.98; I^2^ = 0%); HIIT (MD = −1.34, 95% CI = −6.63, 3.9; *p* = 0.62; I^2^ = 46%).

Influence of different exercise intervention cycles on FEV1 in asthmatic children. More than 8 weeks (MD = 2.89, 95% CI = 1.09, 4.69; *p* = 0.02; I^2^ = 55%); less than 8 weeks (MD = 0.85, 95% CI = 1.45, 3.15; *p* = 0.47; I^2^ = 0%). Influence of different exercise intervention cycles on FVC in asthmatic children. More than 8 weeks (MD = 5.88, 95% CI = −1.30, 10.46; *p* = 0.01; I^2^ = 56%); less than 8 weeks (MD = 0.70, 95% CI = −2.32, 3.71; *p* = 0.65; I^2^ = 78%). Influence of different exercise intervention cycles on PEF in asthmatic children. More than 8 weeks (MD = 4.65, 95% CI = −3.68, 12.98; *p* = 0.89; I^2^ = 0%); less than 8 weeks (MD = −0.27, 95% CI = −3.93, 3.39; *p* = 0.27; I^2^ = 85%).

### 3.2 Effects of exercise on the immune system in children with asthma

Meta-analysis showed that the immune system markers, IL-6 and TNF-α, were significantly reduced in the exercise group. However, exercise did not significantly improve FeNO in asthmatic children: Interleukin-6 (MD = −0.49, 95% CI = −0.81, −0.17; *p* = 0.003; I^2^ = 0%); tumor necrosis factor-α (MD = −0.54, 95% CI = −0.92, −0.15; *p* = 0.006; I^2^ = 0%); Fractional exhaled nitric oxide (MD = −2.45, 95% CI = −11.91, 7.02; *p* = 0.61; I^2^ = 0%) (Table 3).

**TABLE 3:** Effects of exercise on lung function, immune system, body composition, exercise capacity and quality of life in children with asthma.

	Study(*n*)	Participants(*n*)	MD (95% CI)	I^2^ (%)	*p*-value
Lung function					
FEV1	15	642	2.12 (0.70,3.53)	15	0.003
FVC	14	628	2.78 (1.26,4.31)	56	0.0004
PEF	10	513	1.84 (V2.83,6.51)	72	0.44
Immune system					
IL-6	3	132	−0.49 (−0.81, −0.17)	0	0.003
TNF-α	3	132	−0.54 (−0.92, −0.15)	0	0.006
FeNO	3	151	−2.45 (−11.91,7.02)	0	0.61
Body composition					
BMI	6	372	−2.42 (−4.40,0.44)	85	0.02
Exercise capacity					
6WMT	7	320	115.33 (54.64,176.02)	94	0.0002
The Quality of life index					
PAQLQ-Total score	6	370	1.06 (0.46,1.66)	94	0.006
PAQLQ-Emotional	6	370	0.91 (0.76,1.06)	90	<0.00001
PAQLQ-symptoms	6	370	0.87 (0.71,1.02)	95	<0.00001
PAQLQ-activities	6	370	1.20 (0.58,1.82)	93	0.00001

### 3.3 Effects of exercise on the quality of life index in children with asthma

Meta-analysis showed that quality of life (PAQLQ) was significantly improved in children with asthma in the exercise group. PAQLQ-Total score (MD = 1.06, 95% CI = 0.46, 1.66; *p* = 0.006; I^2^ = 94%); PAQLQ-Emotional (MD = 0.91, 95% CI = 0.76, 1.06; *p* < 0.00001; I^2^ = 90%); PAQLQ-symptoms (MD = 0.87, 95% CI = 0.71, 1.02; *p* < 0.00001; I^2^ = 95%); PAQLQ-activities (MD = 1.20, 95% CI = 0.58, 1.82; *p* = 0.00001; I^2^ = 93%) (Table 3).

### 3.4 Effects of exercise on motor ability and body composition in children with asthma

Meta-analysis showed significant improvements in body composition in the exercise group. BMI (MD = −2.42, 95% CI = −4.40, 0.44; *p* = 0.02; I^2^ = 85%) (Table 3). Subgroup analysis showed that aerobic exercise significantly improved BMI in children with asthma, and exercise intervention for less than 8 weeks significantly improved BMI in children with asthma (Table 4).

**TABLE 4:** Subgroup analysis.

	Subgroup		Study	95% CI	*p*-Value	Chi-squared (I2) (%)
FEV1	Sport	Swimming	4	5.98 (0.85,11.11)	0.02*	0
		Aerobic exercise	5	2.89 (0.58,5.19)	0.01*	57
		Ball	3	0.79 (−3.94,5.51)	0.74	0
		HIIT	2	−0.95 (5.83,3.92)	0.70	0
FVC	Sport	Swimming	4	11.07 (6.33,15.82)	<0.00001*	24
		Aerobic exercise	3	5.06 (1.57,8.54)	0.004*	30
		Ball	3	0.77 (−3.88,5.43)	0.74	0
		HIIT	2	0.80 (−4.28,5.89)	0.76	46
PEF	Sport	Swimming	2	2.49 (−7.80,12.78)	0.64	0
		Aerobic exercise	2	0.61 (4.26,5.49)	0.8	0
		Ball	3	0.06 (−5.94,6.07)	0.98	0
		HIIT	2	−1.34 (−6.63,3.95)	0.62	0
BMI	Sport	Aerobic exercise	3	−3.12 (−5.58,-0.66)	0.01*	78
		HIIT	2	−0.06 (−5.58,-0.66)	0.96	43
FEV1	Time	≤8 weeks	10	0.85 (1.45,3.15)	0.47	0
		>8 weeks	5	2.89 (1.09,4.69)	0.002*	55
FVC	Time	≤8 weeks	9	0.70 (−2.32,3.71)	0.65	78
		>8 weeks	5	5.88 (−1.30,10.46)	0.01*	56
PEF	Time	≤8 weeks	7	−0.27 (−3.93,3.39)	0.27	85
		>8 weeks	3	4.65 (−3.68,12.98)	0.89	0
BMI	Time	≤8 weeks	3	−2.99 (−5.70,-0.28)	0.03*	77
		>8 weeks	3	−1.90 (−5.15,1.35)	0.25	85

Effects of different exercise modes on BMI in asthmatic children. Aerobic exercise (MD = −3.12, 95% CI = −5.58, −0.66; *p* = 0.01; I^2^ = 78%); HIIT (MD = −0.06, 95% CI = −5.58, −0.66; *p* = 0.96; I^2^ = 43%); Influence of different exercise intervention cycles on BMI in asthmatic children. More than 8 weeks (MD = −1.90, 95%CI-5.15, 1.35; *p* = 0.25; I^2^ = 85%); less than 8 weeks (MD = −2.99, 95% CI = −5.70, −0.28; *p* = 0.03; I^2^ = 77%).

The meta-analysis showed a significant improvement in 6WMT in the exercise group. 6WMT (MD = 115.33, 95% CI = 54.64, 176.02; *p* = 0.0002; I^2^ = 94%).

## 4 Discussion

This meta-analysis demonstrated the effectiveness of exercise in improving pulmonary function index (FEV1, FVC), immune system (IL-6, TNF-α, Feno), exercise ability (6MWT), body composition (BMI), and quality of life (PAQLQ) in asthmatic children. Asthmatic children should regularly participate in physical exercise.

### 4.1 Effects on lung function

GINA (Global Initiative for Asthma) recommends exercise therapy as a treatment for children with asthma (Bateman et al., 2008). Exercise is also an integral part of the treatment of children with asthma. Exercise-induced asthma is the main reason why most parents ban their asthmatic children from participating in the exercise (de Valois Correia et al., 2012). The effect of exercise on the lung function of children with asthma has been controversial in the past. However, a growing number of published studies have shown that children with asthma can benefit from regular exercise training (Higgins and Thompson, 2002; Higgins et al., 2011; Cohen, 2013). Our meta-analysis shows that exercise intervention can effectively improve pulmonary function parameters FEV1 and FVC in asthmatic children. This is consistent with Xinggui Wu and coworkers’ conclusion (Wu et al., 2020). FEV1 is an essential index of airway function, which reflects airway patency, airway function, and respiratory muscle strength, and used to evaluate the degree of airway obstruction and lesion in asthmatic patients (Alfrayh et al., 2014). FVC reflected the vital capacity of asthmatic patients and be used to assess whether the patients had dysfunction of ventilation. The increase of FEV1 and FVC indicated that exercise could improve airway ventilation function and alleviate asthma symptoms in children with asthma. Our results reinforce previous findings (Wu et al., 2020). Exercise can effectively improve the lung function of children with asthma, which is of great clinical significance. Our meta-analysis results showed that exercise had no significant improving effect on PEF in asthmatic children. This is inconsistent with Xinggui Wu and coworkers’ conclusion (Wu et al., 2020). Due to age differences in subjects, our study focused primarily on 7- to 12-year-old asthmatic children and did not include adults. This suggests that exercise intervention may have different effects on PEF in patients of different ages.

There is no consensus as to which exercise program is most beneficial for children with asthma. Hence, our study used a subgroup analysis of exercise patterns and cycles. We examined the effects of swimming, aerobic exercise, ball games, and HIIT exercise on lung function in children with asthma. We found that swimming and aerobic exercise significantly improved asthmatic children’s pulmonary function index (FEV1, FVC), while ball and HIIT exercise had little effect on FEV1 FVC in asthmatic children. HIIT and ball games did not significantly improve FEV1. This is inconsistent with the findings of Ertürk et al. (2021), which showed that long-term application of HIIT in patients with asthma can achieve better results in lung function and VO2max. This is probably due to the exclusion of two training structures, SIT and RST, into the HIIT study. A recent study showed that the effects of interval sprint training on airway responsiveness are similar to those of aerobic exercise (Good et al., 2019). HIIT training structure based on intermittent sprint training may be more effective in FEV1 of asthmatic children. Ball exercise did not improve FEV1 significantly, possibly due to the short period of exercise included in the study (all less than 8 weeks) and the undetermined intensity of the exercise. Studies have shown that the average heart rate of children participating in ball exercise is higher than aerobic exercise. Ball exercise intervention significantly improves children’s performance in intermittent exercise and reduces cardiovascular stress during submaximal exercise (Bendiksen et al., 2014). This suggests that the HIIT structure should be given more weight in intermittent sprint training, and the intensity of exercise should be paid more attention to in the future. For the analysis of intervention time, we chose the intervention time of 8 weeks as the node, divided into more than 8 weeks, less than or equal to 8 weeks. By comparing the intervention time, we found that more than 8 weeks of intervention time significantly improved pulmonary function index (FEV1, FVC) in children with asthma. The American Thoracic Society/European Respiratory Association recommends 2-3 exercises per week of 30 min each for at least 8 weeks (Rochester et al., 2015). We suggest that children with asthma choose aerobic exercise and swimming as exercise mode, each 30–40 min, and stick to 8 weeks.

### 4.2 Effects on the immune system

Asthma is characterized by the limitation of variable airflow secondary to airway narrowing, airway wall thickening, and mucus accumulation. Airway narrowing is the result of chronic airway inflammation secondary to plasma extravasation and influx of inflammatory cells (such as eosinophil granulocyte, neutrophil, lymphocyte, macrophages and mast cells), and airway hyperresponsiveness (AHR) is an important physiological feature of asthma (Boonpiyathad et al., 2019). AHR may act directly with TNF-α on airway smooth muscle, or release cysteinyl leukotriene C4, D4 indirectly.

TNF-α is produced mainly by macrophages and mast cells and promotes neutrophil (Brightling et al., 2008). IL-6 is secreted by non-white blood cells. Compared with allergic asthma, IL-6 levels are also influenced by viral infection, obesity and increased intrinsic asthma (Simpson et al., 2013; Berry et al., 2006). There was a negative correlation between the level of IL-6 in saliva and the predicted percentage of FEV1. The increase of serum IL-6 in obese asthma patients was related to the impairment of lung function (Raundhal et al., 2015; Lee et al., 2011). The expression of IL-6 and TNF-α in asthmatic patients is closely related to the severity of asthma symptoms. IL-6 and TNF-α are involved in the inflammatory reaction of asthma (Luyu, 2013). We found that exercise intervention can effectively reduce IL-6 and TNF-α levels in children with asthma. This may be due to reduced proinflammatory cytokine release and increased release of anti-inflammatory cytokines (IL-10) (Brüggemann et al., 2015). Fractional Exhaled Nitric Oxide is closely related to airway hyperresponsiveness and airway inflammation, suggesting that airway inflammatory state is an indicator of asthma inflammation (Ricciardolo et al., 2015). We found that the effects of exercise intervention on FeNo were not significant, as is consistent with Toennesen et al. (2018)’s results. This may be due to a certain overlap of FeNO levels between asthmatic and non-asthmatic children, which does not effectively distinguish the different types of allergic disease population. The effects of exercise on improving FeNo in children with asthma need further confirmation.

### 4.3 Effects on motor ability and body composition and quality of life

Eating Habits and physical activity have a significant impact on the quality of life of children and adolescents. Poor eating habits and low level of physical activity can lead to childhood obesity and affect the quality of life of children (Martins et al., 2021). The global prevalence of obesity has increased over the past 30 years, resulting in an increase in the incidence and clinical manifestations of many respiratory diseases. In the United States, about 19 per cent of children aged 6–11 are obese, and the proportion of severely obese children was 5.2% (Hales et al., 2018). Asthma is one of the most typical diseases associated with obesity, and among obese children, asthma is also exacerbated and poorly controlled, the quality of life is also reduced, and physical activity is severely inadequate or below the minimum physical activity level (Okubo et al., 2016; Holderness et al., 2017; Kupkina et al., 2020). Studies have shown that children with asthma have higher BMI and higher rates of obesity than other children (Glazebrook et al., 2006). Having a high BMI can also reduce the quality of life in children with asthma (Kupkina et al., 2020). Our meta-analysis shows that exercise interventions can be effective in reducing BMI in children with asthma. We have also carried on the subgroup analysis to the movement pattern and the movement cycle. We found that aerobic exercise was more effective than HIIT, and the possible reason may be that HIIT exercise requires higher cardiopulmonary capacity of asthmatic children, and the intensity of HIIT exercise is higher, which leads to poor effect of HIIT exercise intervention in asthmatic children. We also found that exercise cycles of less than 8 weeks were significantly more effective. The reason for this result was that the subjects in Kader (2013) had a higher BMI. When excluded this article, more than 8 weeks’ exercise cycle were found to be effective in improving BMI in children with asthma. Indeed, the heterogeneity between the studies impacts the interpretation of the associations with >8 weeks cycles, which also reminds us to be more cautious in the subsequent studies. At the same time, we found that the quality of life and exercise ability of asthmatic children improved significantly after exercise intervention, and the total score (emotional, symptom, activities) of all four parts of the questionnaire improved significantly, and the walking distance of 6 WMT increased significantly. These results suggest that exercise interventions can reduce BMI, improve quality of life, and improve exercise performance in asthmatic children, and these three trends occur at the same time. This is due to the increase of physical activity in asthmatic children, the decrease of BMI in asthmatic children caused by the increase of energy expenditure, and increased participation brought about by regular physical activity. Subjects’ quality of life scores may also be partially biased, because although we think it is a relatively objective evaluation method, it is also derived from subjects’ subjective evaluation, and studies with large sample size can reduce the bias.

The decrease of BMI and the increase of exercise ability have a good effect on the quality of life of children with asthma (Holderness et al., 2017). A high BMI affects immune cells in adipose tissue, leading to an increase in macrophages, mainly pro-inflammatory phenotypes, and an increase in other pro-inflammatory factors (such as TNF-α and Il-6) in obesity (Bulló et al., 2003). Adipose tissue is an important source of mast cell progenitor cells, and mast cells are the key mediators of allergy (Poglio et al., 2010). These factors may lead to asthma symptoms in children with asthma. Regular exercise over a long period of time can effectively reduce the BMI of asthmatic children and promote the release of anti-inflammatory factors (such as IL-4 and IL-5), thereby reducing the symptoms of asthma (Bantulà et al., 2021). Quality of life is a multidimensional concept that includes physical, psychological, emotional and social wellbeing. The positive effects of sports on children are not only reflected in physiology, but also demonstrated by the improved quality of life in children with asthma. Studies show that the higher the physical activity level of asthmatic children, the better their quality of life (Basso et al., 2013). A number of factors limit the participation of asthmatic children in physical activities: their own limited understanding of the symptoms, parents’ concerns about the risks of physical activity, and family values of physical activity. These factors reduce children’s levels of physical activity and athletic ability (Williams et al., 2008). Studies have shown that exercise does not worsen symptoms in children with asthma, but improves the quality of life in children with asthma (Furtado et al., 2019). All the studies included in this meta-analysis showed a significant improvement in the 6 WMT distance of asthmatic children after exercise intervention, indicating that exercise intervention improved the exercise ability of asthmatic children. Recently, a meta-analysis found that cardiorespiratory fitness (CRF) and muscular fitness (MF) were positively correlated with the quality of life in asthmatic children, and higher CRE and MF could improve the relationship between asthmatic children and their peers and families (Bermejo-Cantarero et al., 2021). Therefore, reasonable and effective exercise can significantly improve the body composition, exercise capacity and quality of life of children with asthma, which can be used as a reference for clinical exercise rehabilitation.

### 4.4 Limitations

This study has certain limitations. First of all, the literature included in the experimental methods was not blindly included, because the way of exercise intervention is difficult to implement double-blind method. Second, the inclusion of the study used different ways of exercise intervention and different cycle, and the difference of exercise time may be the source of clinical difference. Finally, although some indexes were based on random effect model, there were still some heterogeneities between some studies.

## 5 Conclusion

Exercise intervention can effectively improve the pulmonary function index (FEV1 and FVC) and the immune system (IL-6 and TNF-α) of asthmatic children, improve the quality of life and exercise ability of asthmatic children and effectively reduce the BMI of asthmatic children. We found that swimming and aerobic exercise were more effective in helping children with asthma than other types of exercise, with a duration of at least 8 weeks, 2 to 3 times per week, and 40–60 min of exercise each time.

## Data Availability

The raw data supporting the conclusion of this article will be made available by the authors, without undue reservation.
